# Clinical, Molecular- and Cytogenetic Analysis of a Case of Severe Radio-Sensitivity

**DOI:** 10.2174/138920212802510475

**Published:** 2012-09

**Authors:** K.M. Greulich-Bode, F. Zimmermann, W.-U. Müller, B. Pakisch, M. Molls, F. Würschmidt

**Affiliations:** 1 Klinik und Poliklinik für Strahlentherapie und Radiologische Onkologie, Klinikum Rechts der Isar, Technische Universität München, Germany; 2 Genetik der Hautcarcinogenese, Deutsches Krebsforschungszentrum, Germany; 3 Institut für Radioonkologie, Medizinische Radiologie, Universitätsspital Basel, Switzerland; 4 Institut für Medizinische Strahlenbiologie, Universitätsklinikum Essen, Germany; 5 Abteilung für Radioonkologie und Strahlentherapie, Landesklinikum Wiener Neustadt, Austria; 6 Radiologische Allianz Hamburg, Strahlentherapie, Hamburg, Germany

**Keywords:** Radiation therapy, DNA damage and repair, Double strand breaks, Comet assay, Radiation induced chromosomal aberrations, M-FISH.

## Abstract

In radiotherapy the normal tissue reaction is often a limiting factor for radiation treatment. Still there is no screening method, which predicts normal tissue reaction on radiotherapy, especially in comparison to tumor tissue, and therefore allows tailoring of the radiation dose to each patient. Here, we present a case of severe radiation-related side effects. We applied classical cytogenetic techniques (Giemsa-banding and staining of centromeric regions), the comet assay as well as multicolor fluorescence *in situ* hybridization on peripheral blood lymphocytes of this patient in order to determine the radio-sensitivity on the DNA level and to correlate these findings with the clinical outcome. Our investigations revealed abnormalities on chromosome 9, deficiencies in the DNA-repair capacity after radiation exposure and a high number of radiation induced chromosomal aberrations. A detected high amount of residual damage two or three hours after radiation exposure and repair as well as the high number of chromosomal aberrations (ChAs) suggests a correlation between repair capacity and radiation induced ChAs. We concluded that the detected abnormalities might serve as a genetic basis for the radio-sensitive phenotype of this patient. Taken together this report strengthens the idea that intensive DNA genomic analysis of individual patients can serve as the basis for more favourable treatment of cancer patients.

## INTRODUCTION

The acute and late radiation related morbidity influences the patients quality of life in radiotherapy and it is hard to treat [1]. It is possible, that the difference in the intrinsic radio-sensitivity predisposes individuals to develop more severe side effects. About 30 % of patients with acute gastrointestinal side effects develop moderate to severe late toxicity effects [2]. More recently, it has been reported that also heart disease after radiotherapy is becoming increasingly clinically important as a late side effect [3]. Unfortunately, no predictive assay is available till now allowing the analysis of the individual radio-sensitivity of patients or the predisposition for side effects. 

It has been proposed that the correlation between phenotype (clinical parameters) and genotype of radiotherapy patients may lead to the identification of parameters enabling the evaluation of such a predictive assay [4]. Since DNA is a primary target of radiation, it seems feasible that molecular genetic approaches can lead to the development of an appropriate prescreening method.

Various modern molecular genetic approaches seem suitable for evaluation of such a screening: The comet assay [5, 6] allows the detection of early and late repair capacity or fluorescence *in situ* hybridization (FISH) techniques [7]. FISH using two or three differently labeled chromosome-specific DNA probes ("chromosome painting") is a commonly used technique to detect stable and unstable chromosomal aberrations [8-14]. FISH has also been used to evaluate predictive assays to determine the intrinsic radio-sensitivity of radiotherapy patients [15]. In 1998 the conventional FISH technique was improved by extending the number of chromosomes to 24 [16], which can be detected simultaneously within one metaphase spread, applying multicolor spectral karyotyping (Sky, 14) or multi-color fluorescence *in situ* hybridization (M-FISH, [17]). The application of M-FISH on radiation induced chromosomal aberrations was shown to be a powerful tool [18-20] and has even led to a specific nomenclature (mPAINT, [21]). 

Here, we present a rare case of severe radiation-related side effects (grade IV CTC/RTOG score). Compared with data from large clinical trials on rectal cancer with hypofractionated schedules using even higher single doses (5.0 Gy instead of two-times 2.5 Gy per day) [22, 23], this toxicity was unexpected from the clinical radiation and the radiobiology view. 

We applied Giemsa-Trypsin-Giemsa (GTG-) and centromere (C-) banding, the comet assay as well as M-FISH on peripheral blood lymphocytes of this patient to determine the radio-sensitivity of this patient on the DNA level. We found evidence that the unexpected severe radiation-related side effects the patient suffered from are based on the genotypic characteristics as specified in our case.

We strongly believe that this report nicely shows that intensive DNA genomic analysis can serve as an important tool leading to personalized medicine as it has been postulated for example among others for pharmacogenetics [24].

## MATERIAL AND METHODS

### Case

We investigated a case of a 51-year-old white male patient suffering from a rectal carcinoma (cT2NxM0). A few months prior to his oncologic treatment, the patient was diagnosed with diabetes mellitus. The family history revealed that his father had been successfully treated for a colorectal carcinoma (died at the age of 85 years) and his mother had suffered from diabetes mellitus (died at the age of 78 years).

The patient’s rectal carcinoma was treated in a neoadjuvant setting with an accelerated fractionation schedule of 2.5 Gy bid (minimum 6 hours of interfraction time interval) to 25 Gy in 5 days within a prospective multicenter protocol. The schedule based on the Swedish experiences, but was split into 2 fractions per day to avoid severe late toxicity [25]. The dose was delivered with 15 MeV photons in a conformal 4-field box technique with adequate dose distribution fulfilling the criteria of ICRU 50 [26]. 

### Comet-Assay

Unstimulated peripheral blood of the patient was taken, cells isolated by the Ficoll-Hypaque technique and used for irradiation experiments investigating the repair capacity of blood lymphocytes. The comet assay was performed as described earlier [6]. The analysis of the comets was done as described in detail in Böcker *et al.* [5]. Briefly, lymphocytes were irradiated *in vitro* with 2 Gy X-rays on ice and either transferred immediately to a slide and embedded in agarose or kept for definite time intervals (up to 3 hours) at 37 C to allow repair and then embedded in agarose on a slide. After lysis of all non-DNA material, a weak electric field was applied which causes the formation of a ‘comet-tail’, dependent on the amount of DNA damage. After staining with ethidium bromide, the ratio of the amount of DNA in the comet tail to the amount in the comet head was determined using a self-designed image analysis system.

As controls 35 normal, 5 “radiation-resistant” (i.e. healthy individuals with an efficient repair) and 5 “radiation-sensitive” individuals (i.e. healthy with a poor repair capacity) were studied and their median repair capacity compared to the repair capacity of the patient.

### Lymphocyte Cultures for M-FISH and Classical Cytogenetic Techniques

Peripheral blood of the patient was taken 11 months after ration treatment and used for investigating the radio-sensitivity of the patient’s blood lymphocytes. Therefore, one heparinized blood sample was irradiated *in vitro* with 3 Gy (photons, 6MeV, 2.8 Gy/min, Siemens MX-2, Germany). A second blood sample served as an “unirradiated” control. Short-term cultures were set up from both blood samples and cultivated for 72 hours. Metaphases were prepared using a standard protocol (see also [18]).

### Classical Cytogenetic

Classical GTG- and C-Banding were performed on the metaphase preparations of the non-in-vitro irradiated samples using standard protocols in order to determine the genetic background of the patient [27].

### M-FISH Experiments

We applied M-FISH [17] to metaphase spreads from the short-term cultured lymphocytes as described earlier [18], applying the SpectraVysionTM System (Vysis plc., Downers Grove, IL, USA). M-FISH was performed as described in the manufacturer's manual. The hybridized metaphase spreads were analyzed with an automated fluorescence microscope (Axioplan 2, Zeiss, Göttingen, Germany) equipped with appropriate filter sets. Images were recorded with a CCD camera (Sensys, Photometrics, Tucson, AZ, USA) and analyzed with the Quips SpectraVysionTM Software package (former Vysis now Applied Imaging).

We analyzed 90 metaphases of the *in vitro* irradiated lymphocyte culture and 93 metaphases of the non-additionally irradiated cultures prepared from the patient’s blood samples. The number of aberrant metaphases was recorded as well as the number of chromosomes involved in aberrations within each abnormal metaphase. This data was compared to the analysis of 3Gy *in vitro* irradiated blood samples of 5 different healthy donors, e.g. in total 529 metaphases.

Additionally, a detailed qualitative analysis of the ChAs was performed, e.g. the frequency of involvement in chromosomal aberrations analyzed for each chromosome.

## RESULTS

### The Patient Unexpectedly Developed Severe Radiation-Related Clinical Side-Effects

The patient was subjected to radiotherapy for treatment of a rectum carcinoma (cT2NxM0). No acute side effects were observed during radiotherapy (25 Gy total). Three days after the last fraction an anterior total mesorectal resection of the primary and regional pelvic lymph nodes was performed. The clinical perioperative course was regular besides discrete diarrhea. Two days after discharge from hospital the patient complained about anal pain. 3 weeks later, intermittent loose bowel and hematochezia could be ascertained. An endoscopic examination was done four weeks after tumor resection revealing ulcerating colitis of the rectum. A biopsy from the rectum showed mucosa of the colon with severe colitis. A recurrent tumor could be excluded. A conservative therapy with steroidal and non-steroidal antiphlogistics was initiated, and a protective anus praeter of the transversal colon was applied after several weeks of unsuccessful conservative treatment. Gradually, the condition of the patient improved. Endoscopic examinations confirmed regeneration of the colonic mucosa. 

11 months after resection the examined patient with Karnofsky Index of 80 % prescribed pain of the lower back radiating into both hips and thighs, intensified by knocking, and a rapidly progressive impotence, which was complete within 9 months. A strong motoric weakness of both lower extremities and signs of a peripheral polyneuropathy (no reflex of the Achilles tendon, reduced reflex of the patellar tendon, a reduced discrimination of peaked and dull) could be seen during the examination. Lower extremities showed post-thrombotic alteration. Further vascular status was normal. Skin was completely inconspicuous even in the originally irradiated areas. Neurologic status of central nerval system was normal with the exception of positive bilateral Lasegue. Internal status (heart, liver, kidneys, lung) was completely regular. Endoscopy of the rectum demonstrated a deep ulcer, hardly healing.

Taken together, the patient was suffering from severe late radiation-related clinical side effects, which were not expected from the radiotherapy scheme, compared with data from former randomized clinical trials [22, 23]. Therefore, they were clinically interpreted as resulting from individual radio-sensitivity.

### The Patient’s Lymphocytes are Deficient in Late Repair Capacity

To determine the repair capacity of the patient’s peripheral blood lymphocytes as a model system for all somatic cells, we applied the comet assay [5, 6]. In Fig. (**1**) the repair kinetics of additionally *in vitro* irradiated lymphocytes of the patient in comparison to *in vitro* irradiated lymphocytes of healthy controls (normal, radio-resistant and radio-sensitive) is shown. During the first hour after X-irradiation with 2Gy (X-rays, 240 kVp) no differences were found between the repair capacity of the patient’s lymphocytes as compared to lymphocytes of healthy control persons. However, during the next two hours almost no further damage repair was detectable in the patient’s lymphocytes, whereas in the control group a second slower component of the repair kinetics can be observed. The residual damage in the patient three hours after irradiation is comparable to the residual damage of the five most radio-sensitive control persons. Therefore, the patient’s lymphocytes were shown to be deficient in late repair capacity.

### Classical Cytogenetic Analysis Revealed Chromosomal Abnormalities in both Copies of Chromosome 9

By GTG- and C-banding on metaphase spreads from peripheral lymphocytes, we identified a strong polymorphism, e.g. a huge heterochromatic block in close proximity to the centromeric region, in one copy of chromosome 9 at 9p12. The second chromosome 9 carried a small pericentric inversion (data not shown). Therefore, both copies of chromosome 9 showed karyotypic abnormalities.

The individuals of the control group, which had been enrolled in the M-FISH experiments, no chromosome 9 abnormalities were detected.

### M-FISH Analysis Detects a High Rate of Aberrant Metaphases and Chromosomes

Our M-FISH experiments on the metaphase spreads prepared from peripheral lymphocytes of the patient revealed that 40.9% (38 of 93) of the investigated metaphases carried radiation induced aberrations 11 months after *in vivo* irradiation. These aberrations included dicentric chromosomes, centric and acentric fragments as well as ringchromosomes. 

As shown in one of our previous studies control persons, which had not been treated by radiotherapy, do neither show structural nor numerical aberrations in peripheral blood lymphocytes [18]. 

After additional *in vitro* irradiation with a single dose of 3Gy, 91.1% (82 of 90) of metaphases carried ChAs. The rate of 91.1% aberrant metaphases in the patients lymphocytes is very high compared to a control group of 5 healthy donors where 56-79% (N=529) of metaphases were carrying aberrations [18].

The number of chromosomes per metaphase involved in aberrations was much higher in the additionally irradiated samples, e.g. the complexity of the ChAs was higher see Fig. (**2**). By comparing the complexity of aberrations as indicated by the number of chromosomes involved in aberrant metaphases form our patient with a control group (all *in vitro* irradiated with 3Gy, 72hour cultures etc), the high complexity became even more evident see Fig. (**2**). 

Therefore, our M-FISH experiments revealed a high number of aberrant metaphases and an elevated level of complexity of the ChAs after *in vitro* irradiation of peripheral blood of our patient.

Additionally, we looked at the chromosomes in detail to see if the ChAs were equally distributed over the whole genome, e.g. according to the chromosome size. Interestingly, we detected differences in the quality of chromosomal aberrations persisting 11 months after radio therapy and appearing after *in vitro* irradiation of the blood lymphocytes. Chromosomes 16, 19, 22, X, Y for example were involved in ChAs after acute *in vitro* exposure to additional 3Gy irradiation quite frequently, whereas the non-additionally irradiated cultures showed no involvement of these chromosomes in aberrations see Fig. (**3**). 

## DISCUSSION

Since early as well as late normal tissue reaction after radiotherapy are a limiting factor for cancer treatment, a screening method for predicting the distinct radio-sensitivity of each patient would be beneficial [2, 28], because this would allow tailoring of therapy to individual patients [3, 29]. Therefore, we investigated a case of unexpected severe radiation-induced late effects - as determined by the clinical findings and outcome after radiotherapy - and correlated the clinical findings with molecular biological results.

Compared with data from recent clinical trials, using radio-biologically more risky fractionation schedules, it was not expected to have caused both early and late neurological and intestinal toxicity. Data from former trials showed impairment of sexual function, but only about 2 % degradation of motoric and sensoric nerval function. The fractionation schedule chosen for our patient based on radiobiological expectations to have a by far lower risk with two-times 2.5 Gy instead of one-time 5.0 Gy per day [22, 23]. 

As a model system for the normal tissue reaction of this patient, we applied molecular- and cytogenetic techniques to the *in vivo* (during radiotherapy treatment of the patient 11 months prior to *in vitro* irradiation of lymphocytes) and additionally *in vitro* irradiated lymphocytes (3Gy) of this patient.

In order to analyze the genetic background of the patient, we applied classical cytogenetic techniques and detected abnormalities on both copies of chromosome 9: one chromosome 9 showed a pericentric inversion and the second chromosome 9 a polymorphism in 9p12 [30]. It is known that inversion of chromosome 9 is one of the heterochromatin variants associated with elevated chromosomal instability, increasing congenital abnormalities and even cancer progress, like for example in the Wiedemann-Beckwith-Syndrome [31]. Although our patient does not suffer from the Wiedemann-Beckwith-Syndrome, this might give rise to a genetic component associated with these chromosome 9 abnormalities, which contributes to the radio-sensitive phenotype of the patient. Since chromosome 9 inversions and polymorphisms are regarded as normal variants in our population, it might be beneficial to study more cases of radio-sensitivity screening for chromosome 9 status of the patients.

We also detected abnormalities in the DNA-repair capacity after radiation exposure. One hour after radiation almost no further damage repair was detectable in the patient’s lymphocytes, whereas in the control group a second slower component of the repair kinetic was observed. The residual damage in the patient three hours after irradiation was comparable to the residual damage of the most radio-sensitive control persons. Therefore, it is tempting to speculate that this deficiency in repairing radiation induced DNA damage might be an explanation for the severe side effects this patient was suffering from after *in vivo* irradiation.

M-FISH experiments revealed a high number of remaining chromosomal aberrations 11 months after radiotherapy and a high rate after additional *in vitro* irradiation of peripheral blood of the patient as detected by M-FISH in comparison to healthy control persons ([18]; Fig. (**2**)). The high amount of residual damage two or three hours after repair and the high number of ChAs found 72 hours after *in vitro* irradiation (3 Gy) or 11 months after *in vivo* irradiation (25 Gy) suggests a correlation between repair capacity and radiation induced ChAs. Thus, cells which do not have the capacity for late repair after radiation exposure might have a higher degree of ChAs than cells with a better repair capacity.

It is tempting to speculate that the abnormalities in the repair capacity, the high degree of ChAs and the polymorphisms found on chromosome 9 are correlated to each other. All together they may contribute to additive effects and serve as the genetic basis for the radio-sensitive phenotype of this patient who developed a severe consequential late effect with an early onset 4 weeks after the end of radiotherapy in spite of low total radiation dose, adequate interfraction interval, and optimal radiation technique.

In our patient who was suspected to suffer from radiation sensitivity, we found increased residual DNA-damage and an elevated number of chromosomal aberrations. We and others hypothesize that there is a correlation between *in vitro* radio-sensitivity of lymphocytes and normal tissue toxicity according to clinical symptoms [4]. Additionally, this case provides evidence that there is a correlation between repair capacity of the cells and the radiation induced chromosomal aberrations: a less effective repair capacity might lead to a higher rate of potentially heritable (stable) and non heritable (unstable) aberrations, which can be looked upon as being the “outcome” after DNA damage repair. The differences in the quality of the chromosomal aberrations in terms of the involved chromosomes might be due to either different repair efficiency or different damage tolerance. One feasible explanation for this is the variance in the gene density of chromosomes [32], e.g. same sized chromosomes have the same chance to acquire ChAs during irradiation but due to differences in gene density, these aberrations cannot equally be tolerated. We are aware that the patient carried further risk factors as diabetes, which can influence the tolerance of radiotherapy and postoperative wound healing as well, and that he suffered from peripheral polyneuropathy of unknown origin. Nevertheless, against the background of rapidly progressing symptoms immediately after preoperative radiotherapy and tumor resection, the most probable reason for the patient’s spectrum of symptoms still is radiation therapy.

Therefore, this study might serve as an example for how broadening the knowledge on what and how unwanted side effects in radio therapy evolve, provides prospects for patients and clinicians to tailor radio therapy using technological advances [33] in combination with new diagnostic tools [34], aiming for personalized medicine. 

## Figures and Tables

**Fig. (1):**
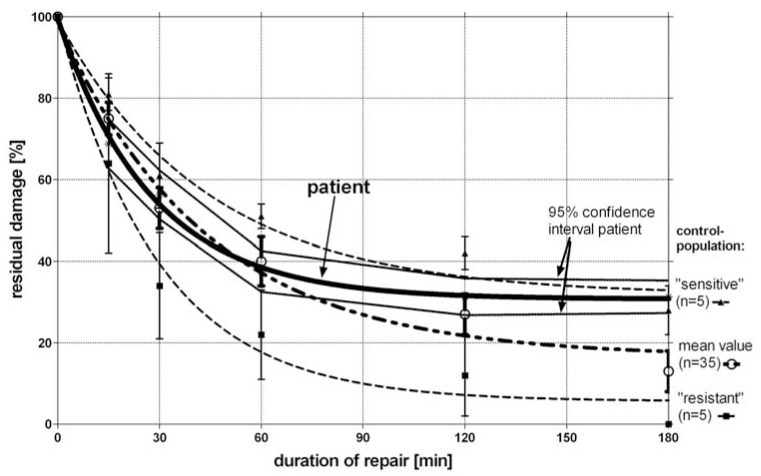
Kinetics of the repair capacity of the patient's lymphocytes in comparison to healthy controls. All lymphocytes were exposed to 2Gy X-rays and investigated up to 180 minutes after radiation exposure. Thick line: repair kinetics of the patient with 95% confidence interval (dotted lines); thick dashed line: healthy individuals with “average” repair kinetics; thin dashed lines: healthy individuals with either poor repair kinetics (upper line) or efficient repair kinetics (lower line); error bars represent 95% confidence intervals. The difference between the mean value and the patient at 180 min after start of repair time is statistically significant at p < 0.05, but not when compared to the “sensitive” healthy individuals.

**Fig. (2):**
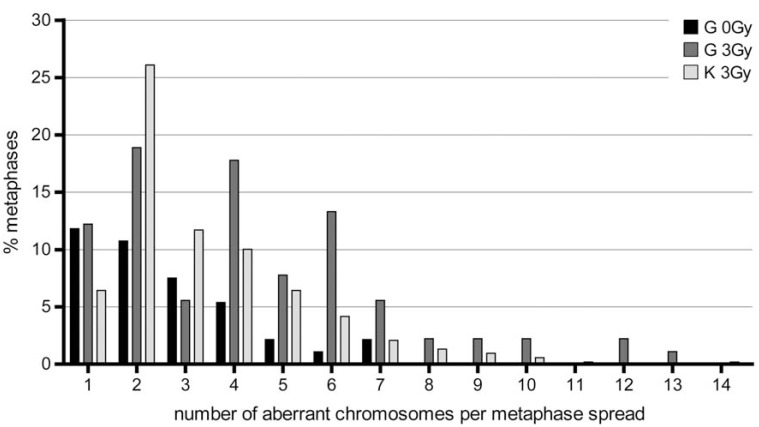
Number of chromosomes involved in aberrations within single metaphases of *in vitro* irradiated lymphocytes (3Gy) of the patient (G; N = 90 = 100%), of a control group (K; five healthy donors; N = 529 = 100%) and the not additionally irradiated (0Gy) lymphocytes of the patient (G; N = 93 = 100%). Please note that non-irradiated blood samples from the healthy control group did not reveal any structural or numerical aberrations (see (18)) and are therefore not shown.

**Fig. (3):**
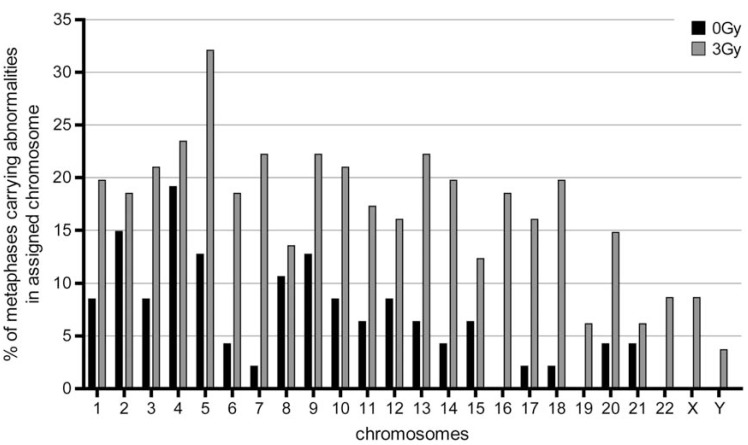
Diagram showing the % of metaphases (lymphocyte cultures) which carry abnormalities in the specific chromosomes a) *in grey* in blood lymphocytes 11 month after radio therapy and b) *in black* in blood lymphocytes 11 months after radio therapy which had additionally be irradiated with an acute dose of 3Gy gamma irradiation *in vitro* (72 hours culture). Interestingly, some chromosomes are no longer involved in aberrations 11 months without *in vitro* irradiation (e.g. chromosomes 16, 19, 22, X and Y).
